# Lymphocyte Activation Gene 3 (LAG-3) Modulates the Ability of CD4 T-cells to Be Suppressed *In Vivo*

**DOI:** 10.1371/journal.pone.0109080

**Published:** 2014-11-05

**Authors:** Nicholas M. Durham, Christopher J. Nirschl, Christopher M. Jackson, Jimmy Elias, Christina M. Kochel, Robert A. Anders, Charles G. Drake

**Affiliations:** 1 Department of Oncology, The Johns Hopkins University School of Medicine, Baltimore, Maryland, United States of America; 2 Department of Pathology, The Johns Hopkins University School of Medicine, Baltimore, Maryland, United States of America; MRC National Institute for Medical Research, United Kingdom

## Abstract

Lymphocyte Activation Gene – 3 (LAG-3) is an immune checkpoint molecule that regulates both T-cell activation and homeostasis. However, the molecular mechanisms underlying LAG-3’s function are generally unknown. Using a model in which LAG-3 blockade or absence reliably augmented homeostatic proliferation *in vivo,* we found that IL-2 and STAT5 are critical for LAG-3 function. Similarly, LAG-3 blockade was ineffective in the absence of regulatory T-cells (Treg), suggesting an important role for LAG-3 in either the responsiveness of conventional T-cells (Tconv) to regulation, or a relative defect in the ability of LAG-3 KO regulatory T-cells (Treg) to suppress the proliferation of Tconv. In this model, LAG-3 KO Treg suppressed proliferation in a manner fairly similar to wild-type (WT) Treg, but LAG-3 KO Tconv were relatively resistant to suppression. Further studies also identified a role for LAG-3 in the induction/expansion of Treg. Finally, we found that LAG-3 blockade (or knockout) led to a relative skewing of naïve CD4 T-cells toward a T_H_1 phenotype both *in vitro* and in *in vivo*. Together, these data suggest that LAG-3 expression on Tconv cells makes them more susceptible to Treg based suppression, and also regulates the development of a T_H_1 T-cell response.

## Introduction

T-cell homeostasis, activation, and survival are tightly regulated processes that generate a pool of cells capable of responding to foreign antigens while retaining the ability to discern self from non-self and preventing autoimmunity [1]. Homeostasis, in particular, is critical in balancing the finite number of lymphocytes that can be maintained in the blood and lymph while allowing adequate “space” for T-cells to expand in response to antigen [2]–[4]. In the periphery, T-cell homeostasis is modulated by at least three processes: 1) TCR engagement, 2) common gamma-chain cytokine signaling, and 3) the suppressive activity of regulatory T-cells (Treg).

Several studies have shown that ongoing, low-level TCR:MHC engagement is required for T-cell survival in the periphery [5], [6]. However, homeostatic stimulation via the TCR is not driven by recognition of a specific cognate antigen, but instead seems to be mediated primarily by interactions between TCR and self-peptides presented by MHC molecules. This interaction provides short-lived TCR signals important in maintaining long-term persistence of peripheral T-cell levels [7]. Indeed, in the absence of TCR interacting with MHC, T-cells fail to survive and numbers decay slowly over time [6]. A second important process that maintains T-cell numbers is signaling via the gamma-chain cytokines IL-7, IL-15 and IL-2 [8], [9]. These molecules signal through a series of specific receptors, each utilizing a common gamma chain (γ_c_), and are necessary for generating functional B-cells, NK cells and T-cells [9]–[11]. Thus, most CD4 and CD8 T-cells require IL-7 for development and maintenance, and expression of the receptor for IL-7 (CD127) is increased on cells destined for a longer-lived memory phenotype [12]–[14]. IL-15, by contrast, may be primarily involved in maintaining numbers of memory CD8 T-cells [15]. Similarly, IL-2 likely serves to control CD4 T-cell levels, including those with a regulatory (Treg) phenotype [16], [17]. As such, genetic depletion of IL-2, or its receptor (CD25) results in a significant reduction in Treg numbers and associated autoimmunity [18]–[20]. Finally, CD4^+^CD25^+^ Treg play an important role in regulating T-cell homeostatic expansion as well as proliferation in response to antigen [21], [22].

Lymphocyte Activation Gene 3 (LAG-3) is an inhibitory T-cell surface molecule that has been found to directly modulate T-cell homeostasis [23], although the precise molecular mechanisms by which LAG-3 performs this function are not known. In terms of TCR signaling, LAG-3 co-localizes with CD8 and CD4 upon TCR engagement and alters TCR signaling [24]. Expression of LAG-3 on T-cells is associated with decreased secretion of cytokines, i.e. an “exhausted phenotype” [25]–[28], although this may not be the case in all models [29], [30]. Finally, we [31] and others [23], [32] found that LAG-3 expression on CD4 T-cells is associated with a Treg phenotype, raising the possibility that LAG-3 blockade or knockout might alter T-cell homeostasis in a Treg dependent manner. In these studies, we sought to determine the pathway(s) by which LAG-3 regulates homeostatic proliferation of CD4 T-cells in a lymphopenic T-cell expansion model. First, we tested a role for IL-2 signaling through STAT5 in the presence or absence of LAG-3 engagement. Next, we tested whether regulatory T-cells are required for LAG-3 to attenuate homeostatic proliferation. Finally, in light of data implicating Treg in LAG-3 function in this model, we tested whether LAG-3 affects the responsiveness of conventional T-cells (Tconv) to suppression by Treg.

## Materials and Methods

### Mice

C57BL/6 RAG1 KO mice and C57BL/6 wild type mice were purchased from Taconic Farms (Germantown, NY). LAG-3 KO mice on a C57BL/6 background were provided by Dr. Yueh-Hsiu Chien (Stanford University, CA) with permission from Dr. Christophe Benoist and Dr. Diane Mathis (Joslin Diabetes Center, Boston, MA). The hemagglutinin (HA) specific TCR transgenic mouse strain 6.5 was provided by Dr. Harald von Boehmer (Harvard University, Boston, MA). The TCR in these animals recognizes an I-E^d^-restricted HA epitope (^110^SFERFEIFPKE^120^] [33]. These mice were backcrossed onto a Thy1.1-congenic B10.D2 background for >12 generations. LAG-3 KO mice were also crossed onto the 6.5 background >10 generations to generate LAG-3 KO 6.5 TCR transgenic mice on a B10.D2 background [31]. FOXP3 DTR mice and FoxP3-GFP mice were a gift from Dr. Alexander Rudensky (Memorial Sloan-Kettering Cancer Center, New York). All animal studies were performed in accordance with protocols (MO13M08) approved by the Animal Care and Use Committee (ACUC) of the Johns Hopkins University School of Medicine.

### Adoptive transfer

Spleens and lymph nodes were harvested, homogenized, and RBC were lysed by ACK solution (Life Technologies Carlsbad, CA) as previously described [29]. CD4 T-cells were purified using Dynal magnetic beads according to the manufacturer’s protocol (Invitrogen Dynal, Norway). CD4 cells were resuspended in 0.2 ml PBS prior to transfer; unless otherwise noted 1E6 cells per mouse were adoptively transferred via retro-orbital injection.

### Antibody treatment

After CD4 T-cell transfer, recipient mice were treated with 200 µg of αLAG-3(Clone C9B7W) [27] or isotype control antibody via intraperitoneal (I.P) injection every other day for 10 days, before spleens and lymph nodes were harvested as previously described [29].

### Flow cytometry

Cells were counted using trypan blue exclusion and stained using the following panel: CD3-APC (Ebioscience, San Diego, CA), CD4-PacOrange (Life Technologies Carlsbad, CA) or CD4-PE (Ebioscience), CD25-FITC (Ebioscience, San Diego, CA). Staining was performed in 50 µl of Facs Buffer (PBS+1% FBS+2 mM EDTA). Cells were washed once and fixed with freshly made 4% paraformaldehyde. Samples were run on a FACSCalibur (BD, San Diego, CA) and data were analyzed using FlowJo software (Treestar Inc. Ashland, OR).

### *In vitro* stimulation and phospho-STAT5 staining

WT and/or LAG-3 KO 6.5 CD4+ T-cells were purified by positive selection (Miltenyi, Germany) using the CD4 positive isolation kit according to manufacturer’s instructions. The CD4 negative fraction was irradiated with 3000 rads and used as antigen presenting cells (APC). For activation, CD4 T-cells were admixed with APC at a ratio of 1∶3 T-cells:APC and stimulated with either 1 or 10 µg of HA Class II peptide for 3 days in the presence of either 50 ug/mL αLAG-3 (clone C9B7W), or an rat IgG1 isotype control antibody (Bioxcell, West Lebanon, NH). 5E5 T-cells were mixed with 1.5E6 APCs in 2 mL of media at 37°C. Detection of phosphorylated STAT5 was performed according to the antibody supplier’s protocol (Cell Signaling Technology, Danvers, MA). Briefly, cells were immediately fixed by placing 1 mL of cell suspension(∼2E6 cells) in 9 mLs of freshly made 4% paraformaldehyde for 30 minutes at 37°C. Next, cells were spun down at 300 g for 5 minutes, decanted, and left on ice for 2 minutes. After chilling the cell pellet, cells were resuspended by slowly adding 90% methanol/10% PBS while vortexing and placed on ice. To complete methanol fixation, cells were chilled at −20°C for 30 min. Next, cells were kept on ice and washed twice with ice cold PBS and spun in a 4°C centrifuge, then blocked with Facs Buffer for 30 minutes. Staining was performed using CD4/FITC (Ebioscience, San Diego, CA) and P-STAT5/AF647 (Cell Signaling). The samples were run on either a FACSCalibur (BD, San Diego CA) or an LSR2 instrument (BD Biosciences, San Jose, CA). Data were analyzed using FlowJo software (Treestar Inc.).

### *In vivo* suppression assay

Untouched CD4 T-cells were isolated using the Dynal negative isolation kit (Life Technologies Carlsbad, CA) according to manufacturer’s instructions, then stained using CD4-APC (Ebioscience, San Diego, CA), CD45RB-PE (BD Biosciences, San Jose, CA), CD25-PE/Cy7 (Ebioscience, San Diego, CA). Following staining, cells were sorted into CD4+CD45RB+CD25−(Tresp) or FOXP3/GFP+(Treg) populations using a FacsAria instrument (BD, San Diego, CA). 1E6 Treg and 4E6 Tresp Cells were transferred into lymphopenic (RAG1 KO) recipients at 1∶4 ratio of Treg:Tresp. On day 7–8 post-transfer, recipient animals were harvested, and single-cell suspensions generated from spleens and lymph nodes as above. For analysis, cells were counted by trypan blue exclusion, and stained for CD3-APC (Ebioscience, San Diego, CA), CD4-PacOrange (Life Technologies Carlsbad, CA) or CD4-PE (Ebioscience), CD25-FITC (Ebioscience, San Diego, CA), followed by analysis using an LSR2 instrument (BD Biosciences, San Jose, CA).

### In vitro suppression assay

Untouched CD4 T-cells were isolated using the Dynal negative isolation kit (Life Technologies Carlsbad, CA) according to manufacturer’s instructions. Splenocytes were depleted of CD4 and CD8 T-cells using Dynal positive isolation kit (Life Technologies Carlsbad, CA) and the negative portion was used as APCs. APCs were irradiated with 3000 rads and pulsed with 1 µg/mL of anti-CD3. 1E5 APCs were added in a 96 well u-bottom plate. WT or LAG-3 KO responders (5E4 cells) were mixed with FOXP3/GFP+ Tregs for a ratio of 1∶1, 1∶2, and 1∶4 Treg:Tresp. These cells were added to the APCs and stimulated for 72 hours prior to pulsing with 0.1 mCi [3H]-thymidine for 16 hours. Finally, cells were harvested onto a glass fiber filter (Perkin Elmer, Waltham, MA), pulsed with scintillation fluid, and read on 1450 Microbeta Trilux (PerkinElmer, Waltham, MA).

### IL-2 ELISA

Approximately 250 µl blood was obtained by cheek bleeding using Microvette CB 300 tubes, then centrifuged at 10,000 g for 5 minutes at room temperature. Serum samples were stored at −80°C. To quantify IL-2 levels, 25 µl of serum was loaded onto a mouse IL-2 Ready-Set-Go ELISA plate (Ebioscience, San Diego, CA), which was developed according the manufacturer’s instructions. Samples were read on a Bio-Tek plate reader (Biotek Winooski, VT).

### Treg induction *in vitro*

CD4 T-cells were purified from LAG-3 KO or WT 6.5 TCR transgenic mice using the Dynal CD4 positive isolation kit according to manufacturer’s instructions. In some experiments, αLAG-3 monoclonal antibody was used at 50 µg/mL in culture. APC’s were obtained from the negative fraction. A total of 5E5 T-cells were admixed with irradiated APCs (3000 Rads) at a ratio of 1∶3 in 24 well plates. Skewing was performed by stimulating cells with 10 µM HA Class II peptide (SFERFEIFPKE) under either T_H_1 conditions or Treg conditions for 3 days. T_H_1 conditions: IL-12 (10 ng/mL), IL-2 (20 ng/mL), anti-IL-4 (200 ug/mL). Treg conditions: TGF-β (5 ng/mL), IL-2 (20 ng/mL), anti-IL-4 (200 ug/mL). To quantify induction, cells were fixed using the Ebioscience FOXP3 transcription factor staining kit. Intracellular staining was performed with the Ebioscience permeabilization buffer, as well as TBET-PE (Ebioscience, San Diego, CA), FOXP3-APC (Ebioscience, San Diego, CA), and IFNγ-PECy7 (Biolegend, San Diego, CA). As above, staining was quantified using a BD LSR2 instrument (BD Biosciences, San Jose, CA) and analyzed using the FlowJo software package (Treestar Inc.).

### Treg induction *in vivo*

1E6 WT or LAG-3 KO Thy1.1+ CD4 T-cells were adoptively transferred into C3-HA Thy1.2+ mice as described above. After 4 days, spleens and lymph nodes were harvested, single cell suspensions prepared, and Treg quantified by staining for: Thy1.1-FITC (Ebioscience, San Diego, CA), CD4-PacOrange (Life Technologies Carlsbad, CA), and CD25-PECy7 (Ebioscience, San Diego, CA). To quantify induction, cells were fixed using the Ebioscience FOXP3 transcription factor staining kit. Intracellular staining utilized the Ebioscience permeabilization buffer with TBET-PE (Ebioscience, San Diego, CA) and FOXP3-APC (Ebioscience, San Diego, CA). As above, staining was quantified using a BD LSR2 instrument (BD Biosciences, San Jose, CA) and analyzed using the FlowJo software package (Treestar Inc.).

### Colitis model

Untouched CD4 T-cells were isolated using the Dynal negative isolation kit and sorted using a FACSAria instrument on CD4+CD45RB+CD25−(Tresp) or CD4+CD45RB−CD25+(Treg) populations using the antibody panel described above. 1E6 Treg and 4E6 Tresp cells were then transferred at 1∶4 ratio of Treg:Tresp into RAG KO recipients [34]. Mice were weighed every other day. On day 50, spleens and lymph nodes were harvested, and Treg quantified via FACS analysis as described above. For histological analyses, colons were removed, fixed by overnight incubation in formalin solution at room temperature, then sectioned and stained with hematoxylin and eosin. To quantify inflammation, sections were scored on a scale of 0–4 by a trained surgical pathologist (R.A.A) in a blinded manner.

### Statistics and Analysis

All data were analyzed using Prism 5 (Graphpad, La Jolla, CA). Groups were compared using unpaired Student’s t-tests.

## Results

### LAG-3 blockade augments homeostatic proliferation *in vivo*

**Figure 1:**
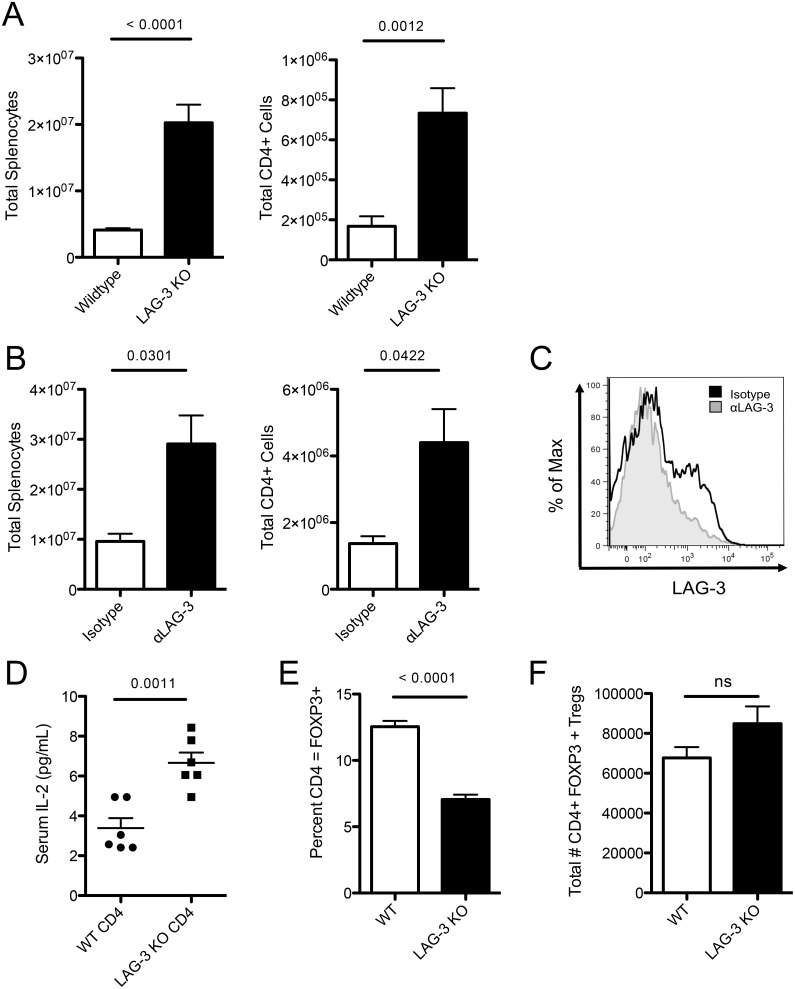
LAG-3 blockade augments homeostatic proliferation *in vivo*. A) 1E6 WT or LAG-3 KO CD4+ T-cells were adoptively transferred into RAG KO mice and harvested on day 10. Splenocytes were then counted and analyzed. B) 1E6 WT CD4+ T-cells were transferred into RAG KO mice. Isotype control antibody or LAG-3 blocking antibody given every 2 days. Splenocytes were then counted and analyzed. C) LAG-3 antibody staining of LAG-3 *in vivo*. D) Serum IL-2 from RAG KO mice with 1E6 WT or LAG-3 KO CD4+ T-cells on Day 7. E) Percentage of CD4+ T-cells that express FOXP3. F) Total number of adoptively transferred CD4+ T-cells expressing FoxP3. Data shown are representative of at least two independent experiments with n = 3–6 mice per group.

To examine the effect of LAG-3 on homeostatic proliferation (HP), WT or LAG-3 KO CD4+ T-cells were adoptively transferred into lymphopenic RAG1 KO mice. After 10 days, total splenocytes were enumerated, and flow cytometry was used to identify CD4+ T-cells. As expected [23], both the total number of splenocytes and the total number of CD4+ T-cells were significantly increased in animals receiving LAG-3 KO T-cells (**Figure 1A**). To verify that augmented proliferation was specifically mediated by LAG-3, WT CD4+ T-cells were transferred into RAG1 KO mice, and animals were subsequently treated with either LAG-3 blocking antibody (αLAG-3) or isotype control antibody. As noted with transferred LAG-3 KO cells, both the total number of splenocytes and the number of CD4+ T-cells were significantly increased in mice treated with αLAG-3 (**Figure 1B**). To confirm *in vivo* LAG-3 blockade, CD4+ T-cells from mice treated with either αLAG-3 or isotype control were stained *ex vivo* with labeled αLAG-3. As seen in **Figure 1C**, staining was substantially blocked in antibody treated mice. Because IL-2 has a well-documented role in augmenting CD4+ T cell proliferation, we next quantified serum levels of IL-2 in recipients of either WT or LAG-3 KO T-cells. As expected, IL-2 levels were significantly increased in animals in which LAG-3 KO cells underwent homeostatic proliferation (**Figure 1D**). Since Treg can play a significant role in controlling HP *in vivo*, we further postulated that the level of FoxP3+ Treg might be decreased in recipients of LAG-3 KO cells, in which HP was augmented. As shown in **Figure 1E**, this was indeed the case; the percentage of FoxP3+ CD4+ T-cells was significantly decreased in recipients of LAG-3 KO cells. Interestingly, the total number of CD4+ FoxP3+ T-cells was not significantly different between recipients of WT CD4+ T-cells and recipients of LAG-3 KO cells (**Figure 1F**), suggesting that LAG-3 may be playing a larger role in the non-Treg CD4+ T-cells. Taken together these data show that LAG-3 attenuates the *in vivo* homeostatic proliferation of CD4+ T-cells, and that augmented proliferation is associated with increased levels of IL-2 and a decreased percentage of Tregs.

### IL-2 and STAT5 are required for LAG-3 blockade to augment homeostatic proliferation

**Figure 2:**
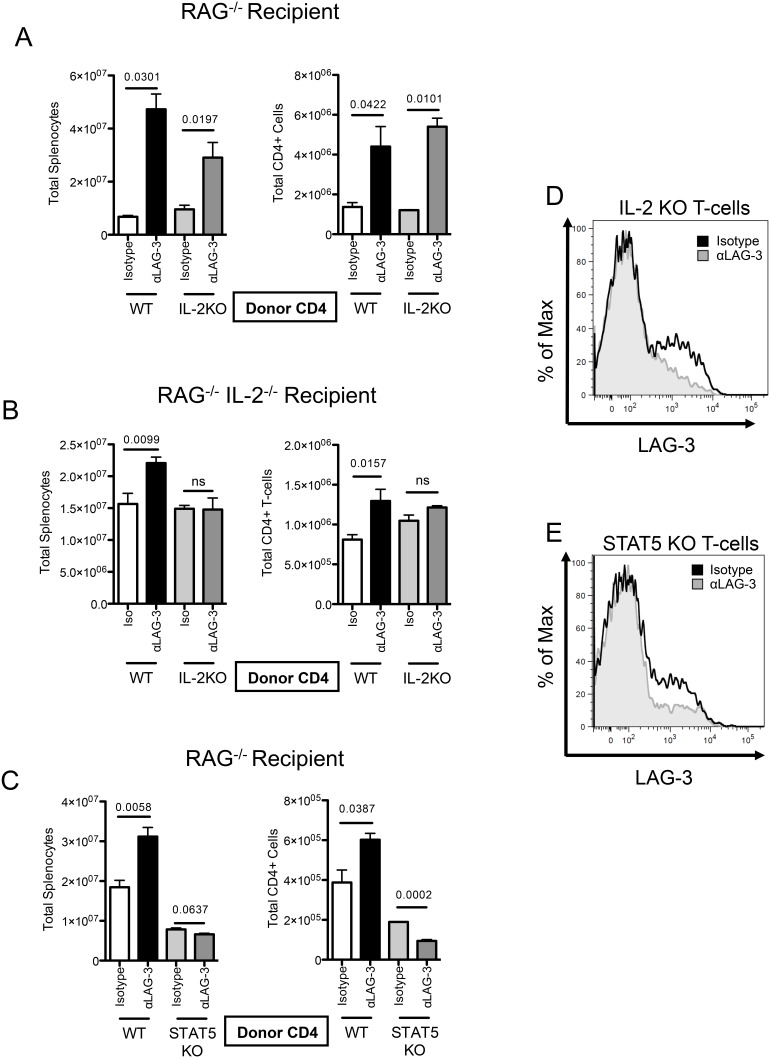
IL-2 is required for LAG-3 blockade to augment homeostatic proliferation *in vivo*. A) 1E6 WT or IL-2 KO CD4 T-cells transferred into RAG KO mice. Isotype control antibody or LAG-3 blocking antibody given every 2 days. Splenocytes counted and analyzed. B) 1E6 WT or IL-2 KO CD4+ T-cells were transferred into RAG KO/IL-2 KO mice. C) 1E6 WT or STAT5 KO CD4+ T-cells transferred into RAG KO mice. Isotype control antibody or LAG-3 blocking antibody was given every 2 days. Splenocytes were then counted and analyzed. D) LAG-3 antibody staining of LAG-3 on IL-2 KO cells *in vivo*. E) LAG-3 antibody staining of LAG-3 on STAT5 KO cells *in vivo*. Data shown are representative of at least two independent experiments with n = 3 mice per group.

Based on the finding that RAG1 KO recipients of LAG-3 KO CD4+ T-cells exhibit increased levels of serum IL-2 as compared to their counterparts receiving WT CD4 cells, we examined whether the increased HP observed in recipients of LAG-3 KO T-cells was dependent upon autocrine IL-2 signaling. To test for such a role, CD4+ T-cells were isolated from either IL-2 KO or WT donors, and adoptively transferred into RAG1 KO mice. Animals were treated every 2 days with αLAG-3 or isotype control antibody; and 10 days after transfer, HP was quantified using flow cytometry. Interestingly, LAG-3 blockade increased the total splenocyte number, as well as the total CD4+ T-cell number, regardless of the ability of transferred cells to secrete IL-2 (**Figure 2A**), arguing against a role for autocrine IL-2 in LAG-3 mediated control of homeostatic proliferation. However, since IL-2 is not totally absent in these animals, it remained possible that exogenous IL-2 (secreted by host cells) was required for LAG-3 to modulate HP. To test whether cell-extrinsic IL-2 is required for LAG-3 blockade to augment HP, we adoptively transferred CD4+ T-cells from IL-2 KO or WT mice into RAG1/IL-2 double knockout (DKO) recipients. In this experiment, DKO mice receiving WT CD4+ T-cells would be exposed only to autocrine IL-2, while DKO recipients receiving IL-2 KO T-cells would have no IL-2 whatsoever. As above, recipient mice were treated with either control antibody or αLAG-3 antibody, and splenocytes were harvested on Day 8. As shown in **Figure 2B**, LAG-3 blockade increased both the total splenocyte number as well as the total CD4 T-cell number in IL-2 KO mice that received WT cells, demonstrating that cell intrinsic IL-2 was sufficient for LAG-3 blockade to augment HP. However, when neither transferred nor host T-cells were capable of secreting IL-2, LAG-3 blockade was completely without effect in terms of HP. To confirm that the lack of effect of blocking antibody was not due to diminished LAG-3 expression, we stained IL-2 KO CD4+ T-cells in the IL-2 KO hosts (**Figure 2D**), and found that LAG-3 was still expressed, and that the blocking antibody successfully blocked staining. Taken together, these data show that LAG-3 blockade requires the presence of IL-2 to augment HP, but that either cell-extrinsic or cell intrinsic IL-2 is sufficient. Because IL-2 is known to signal through STAT5-dependent pathways [35], [36], we next sought to determine whether STAT5 signaling is required for LAG-3 to modulate homeostatic proliferation. To perform these studies, we adoptively transferred WT or STAT5 KO CD4 T-cells into RAG1 KO recipients. These mice were treated with αLAG-3 or isotype control antibody, sacrificed on day 10, and splenocytes were counted and analyzed by flow cytometry. Although, as expected, STAT5 KO CD4 T-cells did not expand to the same degree as WT-cells did, LAG-3 blockade did not augment homeostatic proliferation in the STAT5 KO CD4 T-cells (**Figure 2C**). This was not due to a lack of LAG-3 expression, as STAT5 KO CD4 T-cells still expressed significant levels of LAG-3 (**Figure 2E**). These data suggest that STAT-5 signaling is required for LAG-3 blockade to affect homeostatic proliferation *in vivo*, and that one of LAG-3’s functions may be to modulate the STAT5/IL-2 signaling axis during homeostatic proliferation.

### FOXP3 Expressing Tregs are required for LAG-3 blockade to augment homeostatic proliferation

**Figure 3:**
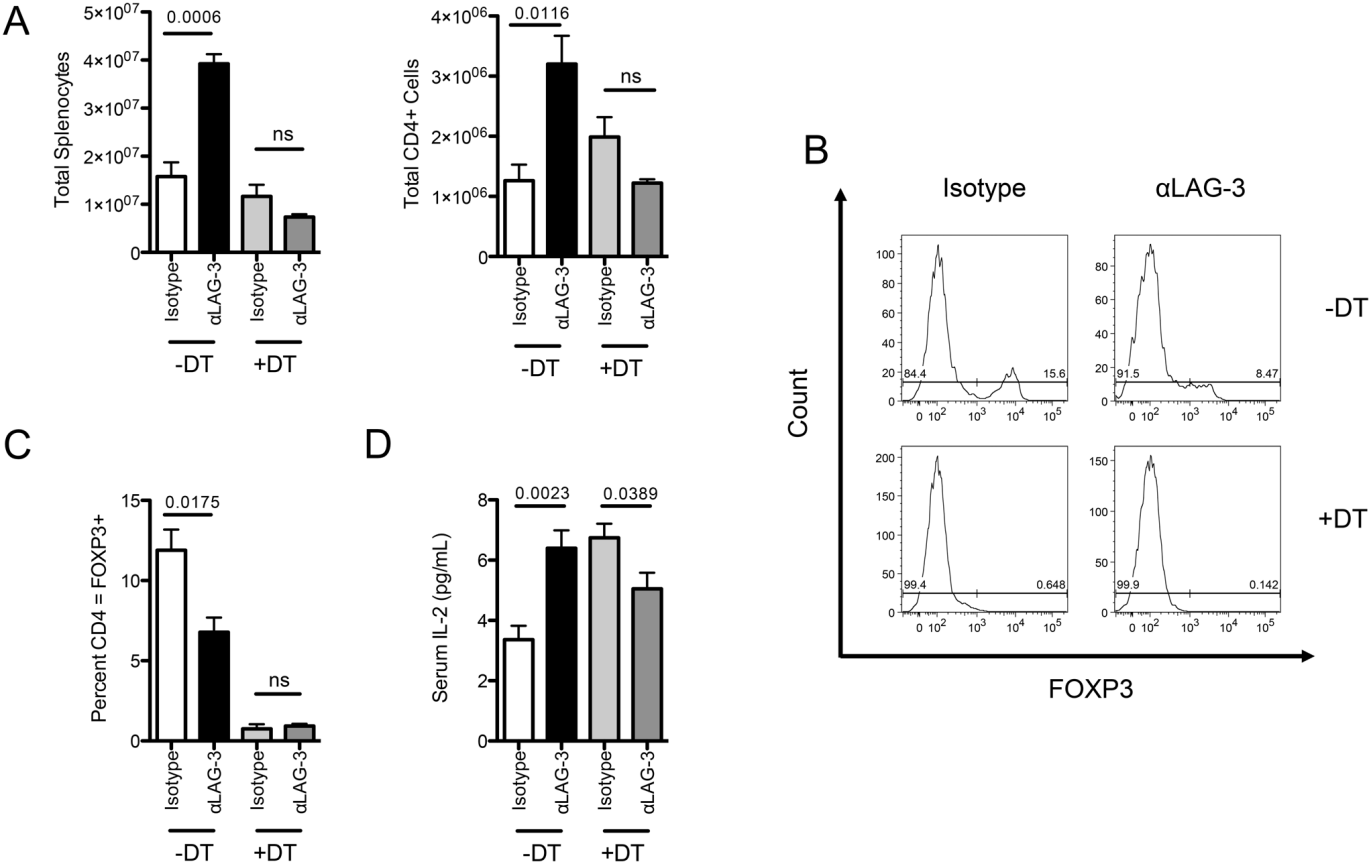
FOXP3 Treg are required for LAG-3 Blockade to augment homeostatic proliferation *in vivo*. A) 1E6 FOXP3 DTR CD4+ T-cells transferred into RAG KO mice. Isotype control antibody or LAG-3 blocking antibody was given every 2 days with either PBS or Diphtheria Toxin. B) FOXP3 expression by flow cytometry ICS. C) Percent of CD4+ T-cells expressing FOXP3 D) Serum IL-2 levels by ELISA. Data shown are representative of at least two independent experiments with n = 4 mice per group.

Because both IL-2 and STAT-5 have been implicated in Treg induction and maintenance [37], [38], we next sought to determine whether FOXP3+ Treg are required for LAG-3 blockade to augment HP *in vivo*. To accomplish this, we utilized FOXP3 DTR mice [39], in which administration of Diphtheria toxin (DT) results in selective ablation of Treg. Here, we adoptively transferred FOXP3 DTR CD4 T-cells into RAG1 KO mice, treated recipient animals with either αLAG-3 and/or DT, and harvested splenocytes on day 10. As shown previously, LAG-3 blockade increased the number of total splenocytes, as well as CD4 T-cells in control animals receiving no DT treatment. These findings mirror those seen in figure 1 using LAG-3 KO CD4 T-cells. When mice were treated with diphtheria toxin to deplete the FOXP3+ cells, we did observe a small, though not significant increase in the total number of CD4+ T-cells. This could be due to the fact that DT depletion will decrease the number of CD4+ T-cells by about 10 percent, or due to the short time course of this experiment (**Figure 3C**). Interestingly, in animals treated with DT, there was no increase in total splenocytes or total CD4 counts upon LAG-3 blockade (**Figure 3A**), suggesting that LAG-3 augments HP in a Treg dependent manner. In addition, the percentage of total CD4 T-cells that were FOXP3 positive was significantly lower in animals treated with αLAG-3 than those treated with isotype control antibody (**Figure 3B–C**), broadly supporting the previously described role of LAG-3 in Treg [31]. In terms of IL-2 levels, depletion of Treg during HP was associated with increased levels of IL-2, but LAG-3 blockade had no further effect on total serum IL-2 beyond the increase due to Treg depletion (**Figure 3D**). Interestingly, the levels of IL-2 in the serum of DT treated mice were similar to those observed in αLAG-3 treated mice in previous experiments. Based on these data, we next tested whether the genetic absence of LAG-3 affects the development of CD4 Treg *in vivo*. Age and sex matched WT and LAG-3 KO mice were harvested, and CD4 Treg from spleen and lymph nodes were quantified for FOXP3 expression. These studies showed no appreciable difference in the percentage of CD4+ T-cells that expressed FOXP3 in either spleen or lymph nodes (**Figures S1A and S1B**). Additionally, these cells expressed similar amounts of other Treg markers such as Helios, GITR [40], [41], and NRP-1 [42]–[45] (**Figure S1C**). Taken together, these data show that LAG-3 blockade requires the presence of Treg to augment HP in vivo.

### LAG-3 KO Treg suppress homeostatic proliferation, but LAG-3 KO responders are resistant to suppression

**Figure 4:**
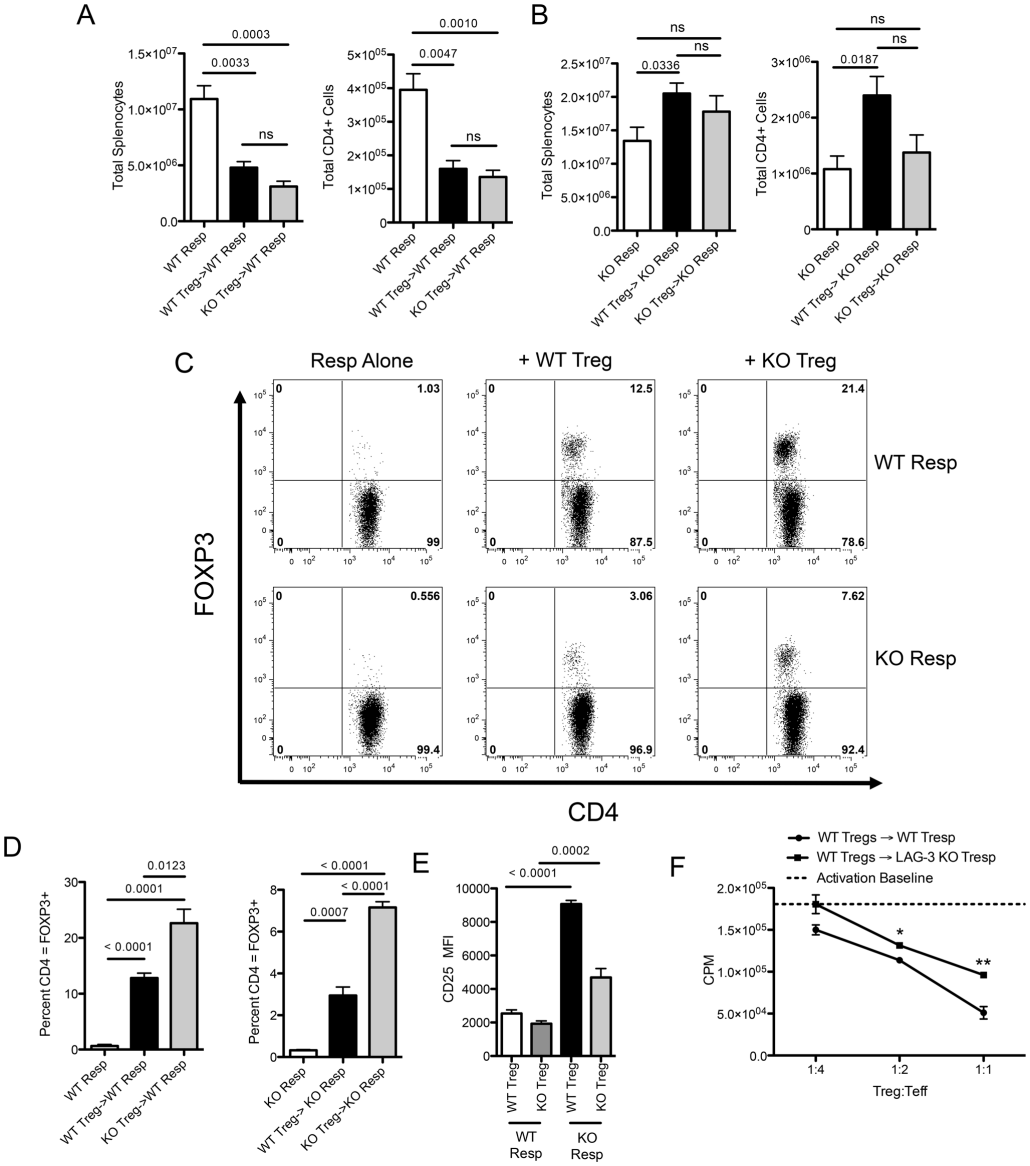
LAG-3 KO Treg suppress homeostatic proliferation, but LAG-3 KO responders are resistant to suppression. A) WT or LAG-3 KO Treg were transferred into RAG KO mice at a ratio of 1∶4 with WT responders. Responders alone received 4E6 WT Tresp with no Treg. B) WT or LAG-3 KO Treg were transferred into RAG KO mice at a ratio of 1∶4 with KO responders. Responders alone received 4E6 LAG3 KO Tresp with no Treg. C) Representative plots of FOXP3 expression in adoptively transferred cells. D) Summary of FOXP3 expression in adoptively transferred cells (n = 4). E) CD25 MFI expression on Treg with either WT or LAG-3 KO Responders. F) WT or LAG-3 KO CD4 Tresp were mixed at a 4∶1 ratio with FOXP3 GFP Treg, stimulated with CD3/CD28, and pulsed with H3-Thymadine after 72 hours. Total CPM counts are shown. Baseline activation of WT or LAG-3 KO Tresp without Treg was not different and is reported by a dashed line. Data shown are representative of at least two independent experiments with n = 4–5 mice per group.

The results described above might be explained by either an inability of LAG-3 KO Treg to suppress HP, or by a relative resistance to suppression in LAG-3 KO responder cells. To distinguish between these two possibilities, we sorted Treg from WT or LAG-3 KO animals (**Figure S2**) and tested their ability to suppress HP by adoptively transferring them into RAG1 KO mice along with WT responders. In this model, WT Treg and Tconv both express similar levels of LAG-3 *in vivo* (**Figures S3A and S3B**). As shown in **Figure 4A**, we found that both WT and LAG-3 KO Treg were capable of suppressing the proliferation of whole splenocytes and total CD4+ T-cells during homeostatic proliferation. Therefore, in this HP model, both WT and LAG-3 KO Treg appeared to be fully functional suppressors. We next investigated the second possibility, i.e. that the absence of LAG-3 on responding cells decreases their ability to be suppressed by Treg during HP *in vivo*. To test this, we adoptively transferred LAG-3 KO responders into RAG1 KO mice, along with either WT or LAG-3 KO Treg. Surprisingly, neither WT nor LAG-3 KO Treg were able to significantly suppress proliferation of LAG-3 KO responders during HP (**Figure 4B**). In fact, the addition of WT Treg actually increased the number of total CD4 T-cells found in the spleen, though the reason for this increase is unclear. These data are further supported by the finding that LAG-3 blockade only increased Tconv cell proliferation, and had no affect on Treg division (**Figures S3C and S3D**). As shown in **Figure 4C** (top row), relative suppression during HP did not correlate well with total FOXP3 levels, as expression of FOXP3 was relatively increased in LAG-3 KO versus WT mediated suppression, despite the similar levels of suppression seen in **Figure 4A**. While there was a trend towards increased suppression with increased FoxP3 expression, this trend was not statistically significant in this model. Similarly, FOXP3 levels were also increased when LAG-3 KO cells were the targets of suppression (**Figure 4C** bottom row), despite their relative inability to be suppressed (**Figure 4B**). Since LAG-3 blockade did not seem to affect Treg cell division (**Figure S3C**), these data suggest that these increases in the expression of FOXP3 are due to increased cell survival. These findings are summarized in **Figure 4D**. We also found that CD25 MFI levels were increased in studies with LAG-3 KO responders (**Figure 4E**), here likely reflecting the relative activation of these cells and consistent with their inability to be suppressed during HP *in vivo* (**Figure 4B**). To further investigate whether LAG-3 KO cells were resistant to suppression, we performed *in vitro* suppression assays, using sorted FoxP3-GFP Treg as suppressors. As shown in **Figure 4F**, LAG-3 KO T-cells were also significantly more resistant to *in vitro* suppression than their wild type counterparts, confirming the *in vivo* results above.

### LAG-3 augments Treg induction *in vitro* and *in vivo*

**Figure 5:**
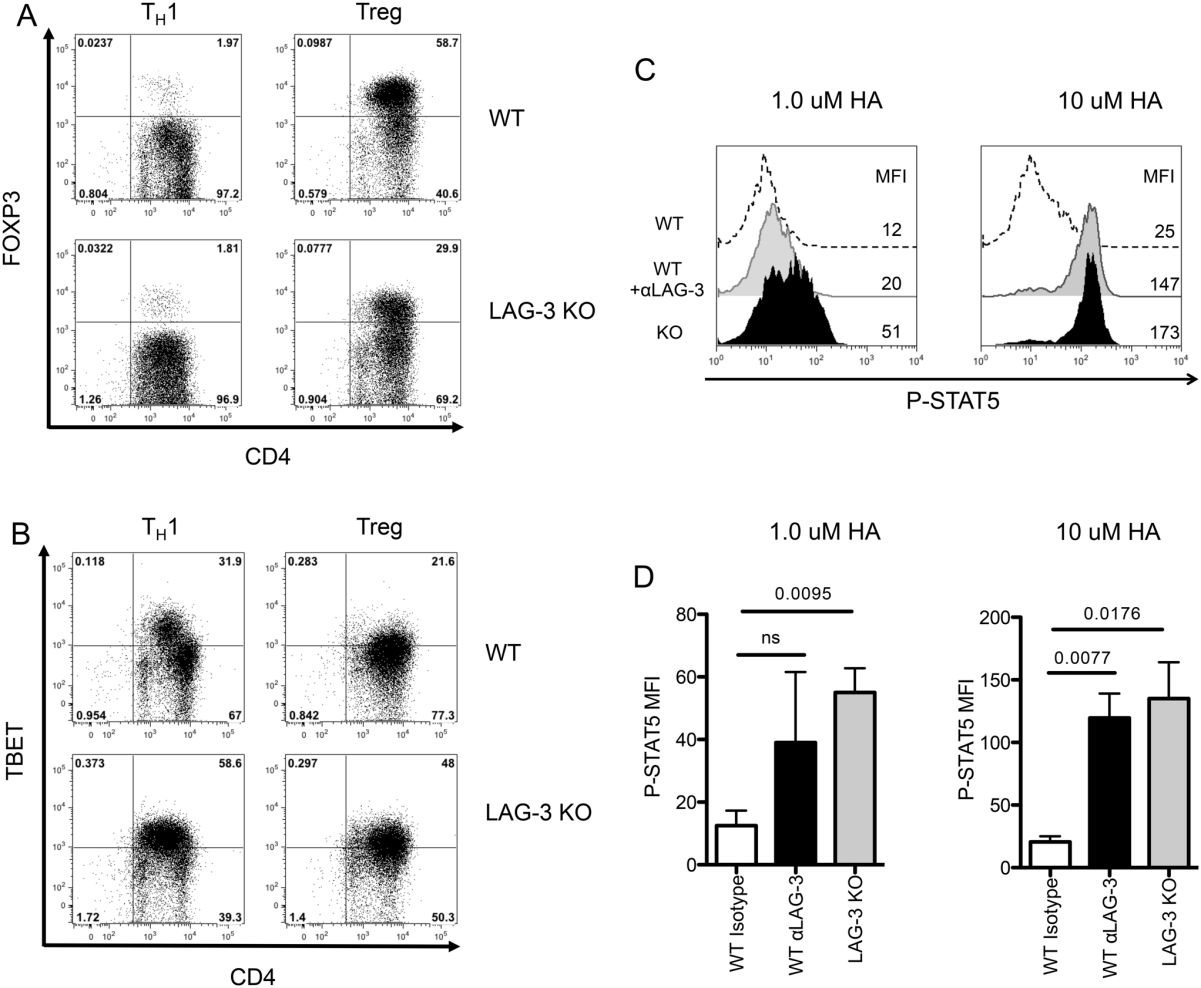
Decreased FOXP3 Treg induction in LAG-3 KO cells *in vitro*. WT or LAG-3 KO 6.5 TCR Transgenic CD4+ T-cells skewed in Th1 or Treg Conditions and analyzed for A) FOXP3 or B) TBET Expression. C) 6.5 TCR transgenic CD4+ T-cells were isolated and mixed with matched splenocytes 1∶3 and stimulated with 1 or 10 µM HA peptide for 3 days. WT or LAG-3 KO 6.5 CD4+ T-cells were treated with αLAG-3 or isotype control antibody at 50 µg/mL and cells were stained for P-STAT5. D) Summary graph of two experiments. Data shown are representative of at least two independent experiments.

**Figure 6:**
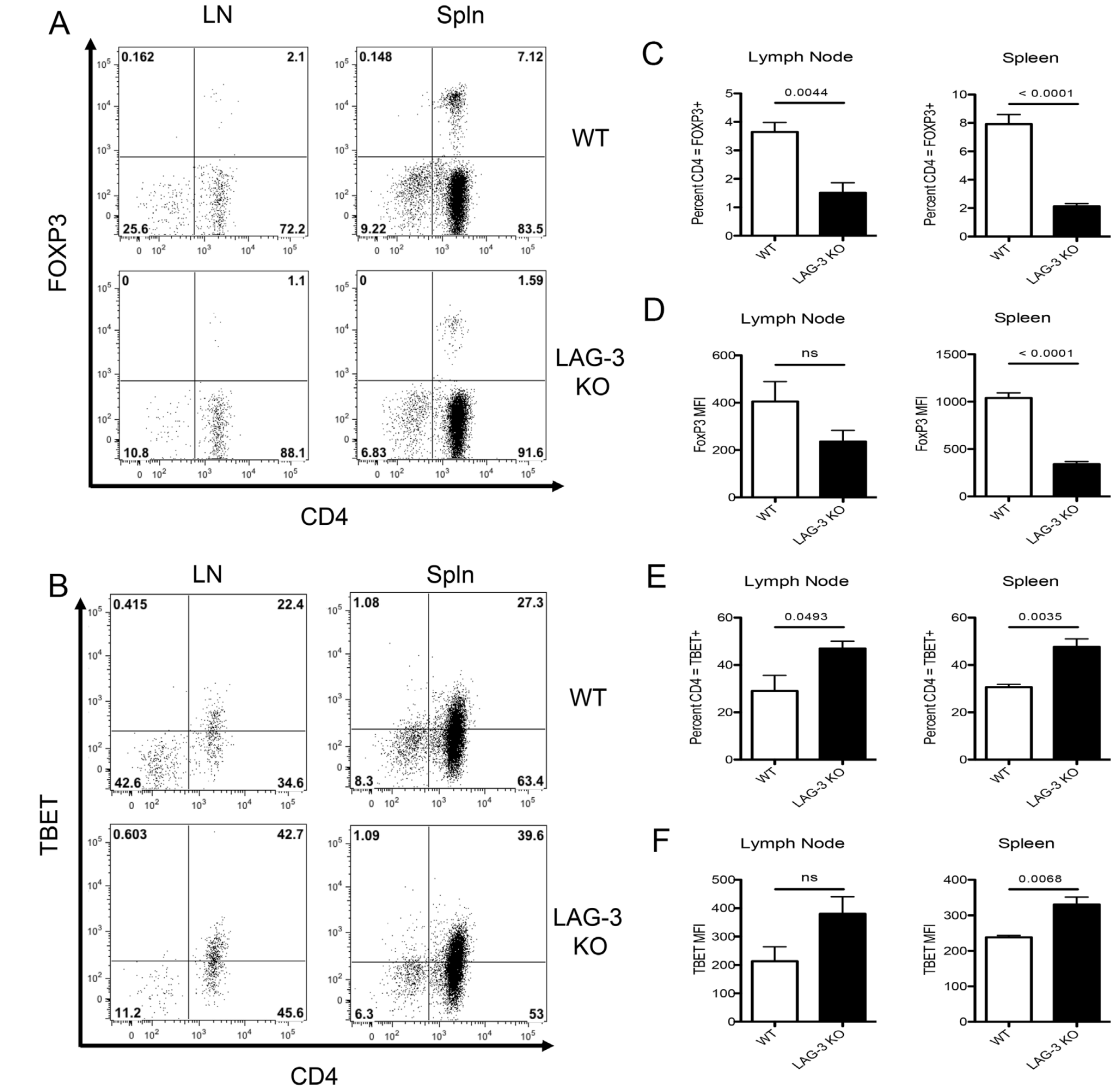
Decreased FOXP3 Treg induction in LAG-3 KO T-cells in an *in vivo* self-tolerance. A) WT or LAG-3 KO 6.5 TCR Transgenic CD4+ T-cells adoptively transferred into C3-HA expressing mice. B) FOXP3 and C) Tbet expression was analyzed and representative graphs shown. C–D) Summary of FOXP3 expression in adoptively transferred cells. E–F) Summary of TBET expression in adoptively transferred cells. Data shown are representative of at least two independent experiments where n = 4 mice per group.

Based on the data shown in **Figure 4**, we hypothesized that LAG-3 signaling could potentially modulate the ability of CD4 T-cells to differentiate toward a Treg versus a T_H_1 phenotype. To test this hypothesis, we performed a set of *in vitro* and *in vivo* studies using antigen-specific CD4 T-cells (6.5 TCR, specific for hemagglutinin (HA)) that were either WT or LAG-3 KO. As a negative control, *in vitro* activation of either LAG-3 KO or WT 6.5 cells under T_H_1 skewing conditions did not result in a significant up-regulation of FoxP3 in either population (**Figure 5A**). However, under Treg skewing conditions, LAG-3 KO cells were far less likely to express FOXP3 than WT cells, demonstrating that LAG-3 likely plays a role in modulating Treg induction/expansion. The relative tendency of LAG-3 KO cells to skew away from a Treg phenotype *in vitro* was accompanied by a corresponding tendency to skew toward a T_H_1 phenotype; as shown in **Figure 5B**, LAG-3 KO CD4+ T-cells were approximately twice as likely as their WT counterparts to express Tbet under T_H_1 skewing conditions. Similarly, LAG-3 blockade during T_H_1 skewing increased production of the T_H_1 cytokine, IFNγ (**Figures S4A and S4B**). We also examined a role for LAG-3 in T_H_2 skewing, but no differences were found in GATA-3 levels in WT versus LAG-3 KO cells (data not shown). Since STAT5 KO CD4 T-cells appeared resistant to LAG-3 mediated augmentation of HP (**Figure 2C**), we queried whether LAG-3 signaling down-regulates phosphorylated STAT5 (pSTAT5) levels. To test this, we stimulated HA-specific CD4+ T-cells *in vitro* and quantified pSTAT5 levels by intracellular staining. As shown in **Figures 5C and 5D**, pSTAT-5 levels were elevated in cells in which LAG-3 was either antibody-blocked, or genetically absent. Taken together these data demonstrate a role for LAG-3 in Treg induction *in vitro*, and that LAG-3 signaling likely functions at least partially via down-regulation of pSTAT5 signaling. We next extended these studies to test whether LAG-3 has a similar role in modulating Treg induction *in vivo*. To perform these experiments, we adoptively transferred antigen-specific WT or LAG-3 KO cells into mice that express their cognate antigen (HA) as a self-antigen (C3-HA). In this model, CD4 T-cells are rendered anergic and acquire regulatory function [46]. This model is different from previous experimental models as the C3-HA mice are not lymphopenic, and therefore, these cells are unlikely to undergo HP as a result of adoptive transfer. As show in **Figures 6A and 6B**, LAG-3 KO CD4+ T-cells were less likely to express FoxP3 and more likely to express Tbet *in vivo*. These data are summarized in **Figures 6C–F**, and, along with the data above, provide evidence the LAG-3 signaling modulates phosphorylation of STAT5 and skews CD4+ T-cells toward a Treg phenotype both *in vitro* and in multiple *in vivo* models.

### LAG-3 KO Responders are Relatively Resistant to Suppression in an *In Vivo* Colitis Model

**Figure 7:**
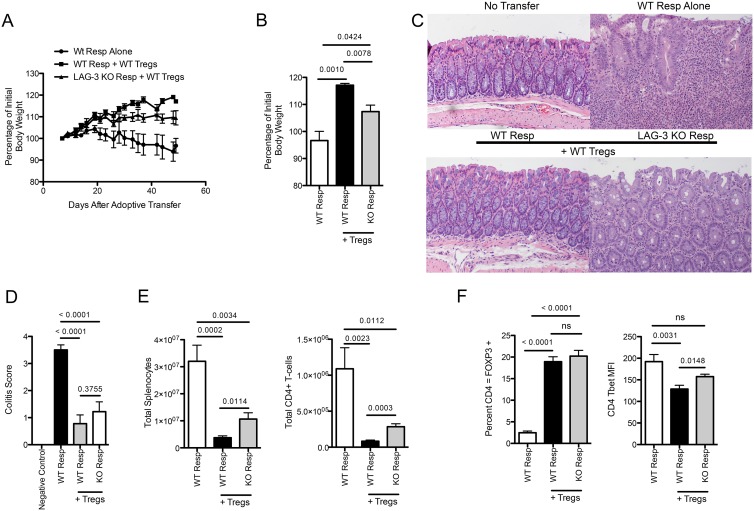
WT Treg Cannot Completely Protect against LAG-3 KO Tresp in a Colitis Model. A) WT or LAG-3 KO Tresp were transferred into RAG KO mice at a ratio of 4∶1 with WT Treg. Mice were weighed 3 times weekly for 50 days. Percentage of initial body weight is reported. B) Percentage of initial body weight at Day 49. C) H & E staining of histological sections of colons from the 4 groups of mice. D) Blinded histological score of colitis in mouse groups. E) Total splenocytes as well as total CD4+ T-cells were counted and analyzed. F) Percentage of CD4+ T-cells that were FOXP3 or TBET positive. Data shown are representative of at least two independent experiments with n = 8–10 mice per group.

To test whether LAG-3 modulates the ability of Tresp to be suppressed in a potentially more physiologically relevant context, we turned to the well-described *in vivo* model of induced colitis [34]. As a positive control we adoptively transferred WT responders into RAG1 KO mice. As shown in **Figure 7A**, these mice progressively lost weight, and upon sacrifice at day 50 had obvious colitis upon pathological examination (**Figure 7C**). As a control for Treg mediated prevention of colitis, WT responders were transferred along with WT Treg. These mice gained weight over the course of the experiment (**Figure 7A**) and showed only mild colitis at the experiments close. LAG-3 KO responder cells, by contrast, were relatively resistant to suppression this model; young mice gained only a minimal amount of weight over the course of the experiment (**Figure 7B**), and demonstrated an intermediate level of colitis upon histological examination (**Figure 7C**). In terms of overall colitis score, mice receiving LAG-3 KO responder cells plus Treg showed slightly more colitis then mice receiving WT responders and no Treg at all, although that increase was not statistically significant (**Figure 7D**). This intermediate phenotype was, however, reflected in overall cell numbers, since, mice receiving no Treg (WT responders only) had greatest total numbers of splenocytes, followed by mice receiving LAG-3 KO responders (+ suppressors), and then by mice receiving WT responders and suppressors (**Figure 7E**). We also tested whether these observed differences in colitis could be explained by an unexpected difference in the relative survival of the WT Treg population, but as shown in **Figure 7F**, the percentage of suppressors appeared to be relatively similar in mice the received either LAG-3 KO or WT responders. Interestingly, and consistent with the skewing data shown in Figures 5 and 6, the LAG-3 KO responders were more likely to display a T_H_1 (Tbet +) phenotype (**Figure 7F**). While these findings do not demonstrate the absolute susceptibility of LAG-3 KO responders, they do demonstrate that the LAG-3 KO responders are relatively more resistant to suppression than their WT counterparts in an *in vivo* colitis model, and confirm our previous *in vitro* results.

## Discussion

LAG-3 has been shown to regulate homeostasis of both lymphocytes and some dendritic cell populations [23], [32], and here we confirmed that blockade of LAG-3 significantly increases homeostatic proliferation *in vivo.* This augmented HP is dependent on the presence of IL-2, although the source of IL-2 does not appear to be especially critical. Additionally, we found that LAG-3 mediated augmentation of HP was dependent on the presence of STAT5, since enhancement of homeostatic proliferation did not occur in mice deficient in both STAT5a and STAT5b. This may be because LAG-3 signaling can directly down-modulate the level of STAT5 phosphorylation in activated T cells, suggesting that LAG-3 signaling is STAT5 dependent, at least in the HP model studied here. Interestingly, similarly to the studies with diphtheria toxin, the addition of LAG-3 blockade to STAT5a/b KO mice decreased the number of splenic CD4+ T-cells, potentially due to either increased CD4+ T-cell death, or due to increased CD4+ T-cell activation in the absence of Treg. Further studies will be necessary to determine the exact cause of this decrease. However, to the best of our knowledge this is the first report showing that LAG-3 signaling directly modulates levels of pSTAT5, although one group previously showed that LAG-3 blockade is able to increase phosphorylation of Akt, which is downstream of the JAK/STAT pathway [47]. Finally, using FOXP3-DTR mice, we found that FOXP3 Treg are necessary for LAG-3 blockade to enhance HP. In the absence of FoxP3+ Treg, LAG-3 blockade had no additional effect on HP. These data are consistent with previous studies, in which LAG-3 has been shown to be an important molecule present on Tregs [23], [31], [48], [49].

Based on those findings, we next tested whether the presence of LAG-3 on Treg was critical in their ability to suppress proliferation, either during HP or in a standard *in vitro* suppression model. Here, we found no appreciable difference between WT and LAG-3 KO Treg in their ability to suppress T-cell proliferation. This finding was supported by the fact that LAG-3 KO and WT Tregs expressed similar levels of Treg associated proteins, such as GITR, NRP-1, and Helios. Instead, we found that LAG3 KO responders were resistant to suppression, both *in vitro* and *in vivo*, and that the addition of WT Treg actually resulted in increases in the number of responders present. These findings are in agreement with a recent publication showing that LAG-3 KO responders are harder to suppress in a murine model of GVHD, and that LAG-3 KO Treg had similar suppressive function to WT Treg [50]. Yet, these findings are also slightly discordant with prior studies showing that LAG-3 KO Treg have diminished suppressive capabilities [23], [30]. However, in one of these studies, the purity of the Tregs being assayed was never explicitly described, and TCR transgenic responders were used (OT-II) [23]. Additionally, in another study claiming a role for LAG-3 on Treg, suppressive function was only described *in vitro*, while the majority of our current studies examined *in vivo* suppressive activity.

The relative resistance of LAG-3 KO responders to be suppressed was observed in three separate models: 1) during HP 2) *in vitro* and 3) in a fairly well established *in vivo* colitis model. Our colitis data further support the notion that LAG-3 KO cells are relatively resistant to suppression, though these experiments cannot examine their absolute potential to be suppressed without other controls. RAG1 KO mice receiving LAG-3 KO responders + Treg displayed a generally intermediate phenotype, i.e. their colitis was less severe than those receiving no Treg at all, but slightly more severe (especially in terms of cell numbers) than mice receiving WT Treg and WT responders. This intermediate resistance to suppression is consistent with the phenotype of LAG-3 KO mice, which show an initial lymphocyte expansion at birth, but which do not develop overt autoimmunity. However, in terms of the objective measurement of body weight, we show that LAG-3 KO responders are more resistant to suppression.

In addition, we showed that LAG-3 blockade (or genetic absence) affects CD4+ T-cell skewing, promoting a T_H_1 phenotype and relatively inhibiting Treg induction. Taken together, these data suggest a model in which LAG-3 modulates CD4+ T-cell homeostasis by two mechanisms. First, LAG-3 signaling modulates T-cell signaling and sensitivity to Treg suppression by limiting signaling through STAT5. Second, LAG-3 signaling increases FOXP3 Treg differentiation. When LAG-3 is blocked, there is a decreased induction of FOXP3+ Treg, resulting in diminished suppression and increased CD4+ T-cell expansion. If no Treg are present, then LAG-3 blockade generally drives naïve CD4+ T-cells toward expression of T-bet and T_H_1 differentiation.

These data are important in understanding the potential mechanism of action of LAG-3 blockade, since human anti-LAG-3 is currently being evaluated in a Phase I clinical trial in patients with advanced cancer (Clinicaltrials.gov ID: NCT01968109). The data presented here suggest that LAG-3 blockade could possibly affect CD4+ T-cell populations in treated patients, skewing away from a Treg phenotype and affecting the ability of CD4+ T-cells to be suppressed. One interesting aspect of the current trial is that LAG-3 blockade will eventually be tested in combination with PD-1 blockade, a regimen that has demonstrated synergy in tumor, autoimmune, and infectious disease models [51]–[53]. Based on the relative absence of autoimmunity in LAG-3 KO mice, blocking LAG-3 clinically could potentially result in diminished toxicity as compared to blocking other immune checkpoints like CTLA-4, and might render LAG-3 blockade a reasonable partner in combination immunotherapy studies in cancer and in chronic infectious disease.

## Supporting Information

Figure S1 **WT and LAG-3 KO mice have similar numbers of Treg and similar levels of Treg markers.** A) Representative CD4 FOXP3 Levels. B) Percent of CD4+ T-cells that express FOXP3 in spleen and lymph node. C) NRP-1, Helios, and GITR expression on Treg. Data shown are representative of at least two independent experiments with n = 3 mice per group.
(TIF)

Figure S2 **Post-Sort analysis shows similar purities of Treg for suppression experiments as well as colitis experiments.** Representative pre and post sort data from Tresp and Treg sorts.
(TIF)

Figure S3 **LAG-3 Blockade Augments Tconv Cell Division During Homeostatic Proliferation.** A) 1.5E6 congenically marked Treg were mixed with 6E6 wildtype Tconv before being labeled with 5 µM CFSE and adoptively transferred into RAG KO mice for 7 days. Isotype control or LAG-3 blocking antibody was administered on day 0, 3 and 5 by intraperitonial injection. A) Representative expression of LAG-3 on Tregs and Tconv following homeostatic proliferation. B) Percent of Tregs and Tconv cells expressing LAG-3. C) Representative CFSE dilution of Tregs and Tconv cells following LAG-3 blockade. D) Percent of adoptively transferred cells left undivided following 7 days *in vivo.* Data are representative of at least two independent, similar experiments with n = 4–5 mice per group.
(TIF)

Figure S4 **LAG-3 Blockade Augments Production of IFNγ During** ***in vitro*** **T_h_1 Induction.** WT 6.5 TCR Transgenic CD4+ T-cells skewed in Th1 or Treg Conditions with either isotype control antibody or LAG-3 blocking antibody. A) Representative production of IFNγ. B) Percent of 6.5+ CD4+ T-cells producing IFNγ. Data are representative of triplicate wells.
(TIF)
