# Supplementary figures and images for: Lymphocyte Activation Gene 3 (LAG-3) Modulates the Ability of CD4 T-cells to Be Suppressed In Vivo
Source: PLoS One. 2014 Nov 5;9(11):e109080. doi: 10.1371/journal.pone.0109080 (PMC4220939; doi:10.1371/journal.pone.0109080)

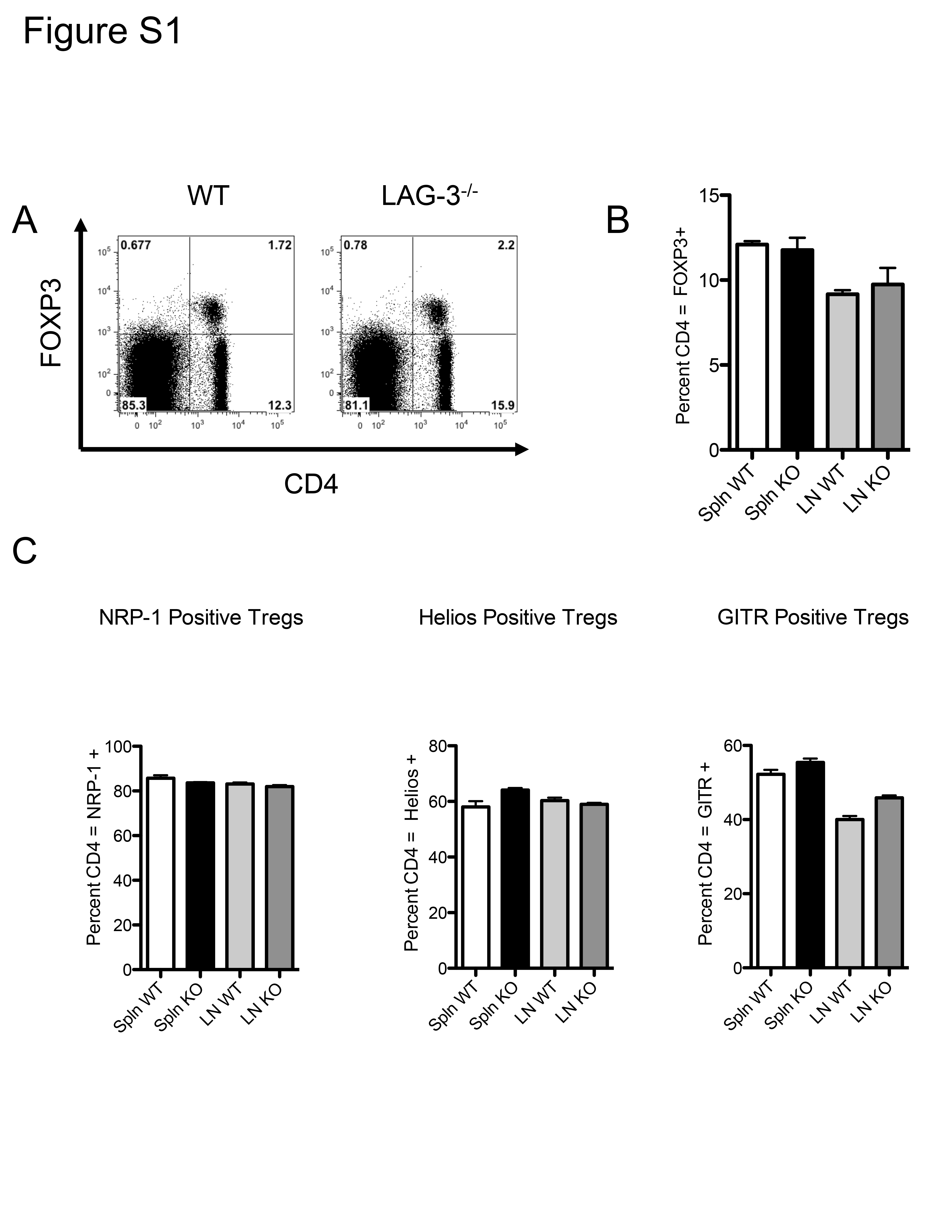

Supplement: Figure S1 — WT and LAG-3 KO mice have similar numbers of Treg and similar levels of Treg markers. A) Representative CD4 FOXP3 Levels. B) Percent of CD4+ T-cells that express FOXP3 in spleen and lymph node. C) NRP-1, Helios, and GITR expression on Treg. Data shown are representative of at least two independent experiments with n = 3 mice per group. (TIF) [file pone.0109080.s001.tif]

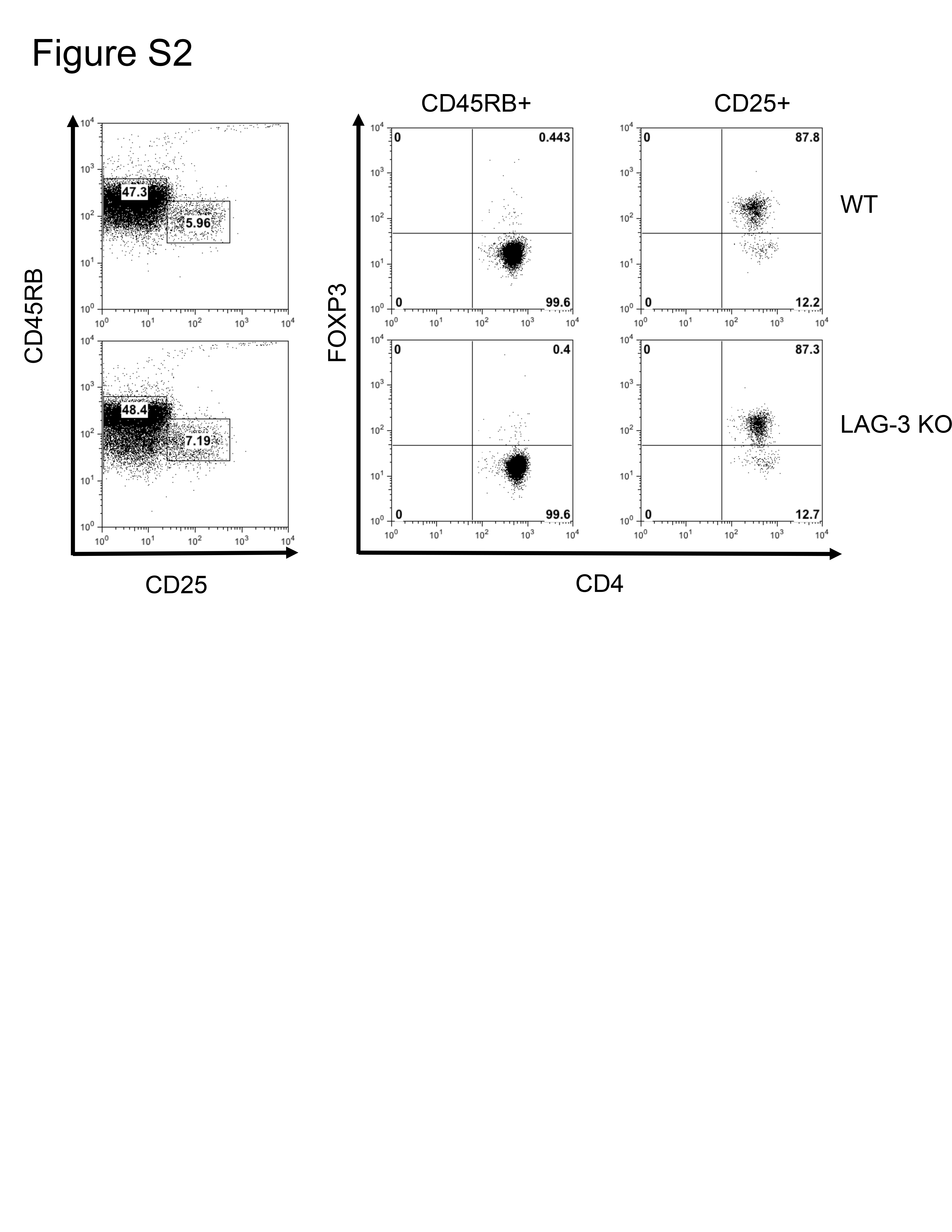

Supplement: Figure S2 — Post-Sort analysis shows similar purities of Treg for suppression experiments as well as colitis experiments. Representative pre and post sort data from Tresp and Treg sorts. (TIF) [file pone.0109080.s002.tif]
